# A Zero-Inflated Latent Dirichlet Allocation Model for Microbiome Studies

**DOI:** 10.3389/fgene.2020.602594

**Published:** 2021-01-22

**Authors:** Rebecca A. Deek, Hongzhe Li

**Affiliations:** Department of Biostatistics, Epidemiology, and Informatics, Perelman School of Medicine, University of Pennsylvania, Philadelphia, PA, United States

**Keywords:** metagenomics, gibbs sampling, zero inflated dirchlet distribution, mixture models, microbial community

## Abstract

The human microbiome consists of a community of microbes in varying abundances and is shown to be associated with many diseases. An important first step in many microbiome studies is to identify possible distinct microbial communities in a given data set and to identify the important bacterial taxa that characterize these communities. The data from typical microbiome studies are high dimensional count data with excessive zeros due to both absence of species (structural zeros) and low sequencing depth or dropout. Although methods have been developed for identifying the microbial communities based on mixture models of counts, these methods do not account for excessive zeros observed in the data and do not differentiate structural from sampling zeros. In this paper, we introduce a zero-inflated Latent Dirichlet Allocation model (zinLDA) for sparse count data observed in microbiome studies. zinLDA builds on the flexible Latent Dirichlet Allocation model and allows for zero inflation in observed counts. We develop an efficient Markov chain Monte Carlo (MCMC) sampling procedure to fit the model. Results from our simulations show zinLDA provides better fits to the data and is able to separate structural zeros from sampling zeros. We apply zinLDA to the data set from the American Gut Project and identify microbial communities characterized by different bacterial genera.

## 1. Introduction

The advent and proliferation of next-generation sequencing (NGS) technologies has given rise to many large-scale high-throughput microbiome studies (Turnbaugh et al., 2007; Gilbert et al., 2014; McDonald et al., 2018). Classical statistical techniques are not able to evaluate such data due to its inherent high dimensional, count-based, and sparse nature. Consequently, novel statistical methods are necessary for accurate and unbiased analysis of such data.

Much of microbiome research has focused on high-dimensional statistical methods, as a single 16S rRNA gene sequencing sample can produce tens of thousands of sequencing reads from hundreds of different amplicon sequence variants (ASVs). Of particular interest are techniques for dimensionality reduction. Commonly used methods include principal coordinate analysis (PCoA) with distance measures, such as weight and unweighted UniFrac distance and Bray-Curtis dissimilarity, or canonical correlation analysis with sparsity assumptions (Chen et al., 2013; Hawinkel et al., 2019). More recently, studies have begun to focus on understanding microbial dynamics within the human microbiome. Single-species analysis, that focus on one species at a time in a “parts-list” fashion, are not able to capture complex and dynamic interactions. These inter-species interactions form the basis of distinct underlying subcommunity structures and failing to account for them contributions to the data heterogeneity commonly seen in microbiome studies. As such, network-based approaches have been successfully applied in this area (Faust and Raes, 2012; Layeghifard et al., 2017). These methods use co-occurrence or correlation measures to identify pairwise interactions in cross-sectional studies (Faust et al., 2012; Friedman and Alm, 2012; Kurtz et al., 2015). Others use temporally conserved covariance to identify interactions in longitudinal studies (Raman et al., 2019).

Generative probabilistic mixture models are able to act as a dimensionality reduction technique while simultaneously describing microbial dynamics via subcommunity identification. When applied to microbiome data the latent variable(s) in a mixture model have meaningful biological connotations. Specifically, they represent distinct subcommunity profiles, or structures, that give rise to the observed samples. The simplest of these is the Dirichlet-multinomial mixture model (Holmes et al., 2012). This model is a generalization of the Dirichlet-multinomial hierarchical model. Rather than assuming that all samples in a cohort are generated from a single community profile, as the Dirichlet-multinomial model does, the mixture model assumes the cohort contains many different subcommunity structures and each of the samples is generated by one of them (Holmes et al., 2012). As such, a sample can be described by its subcommunity assignment rather than a high-dimensional vector of ASV counts. Though, the Dirichlet-multinomial mixture model may still be too restrictive to accurately capture microbial community structures and all the heterogeneity of microbiome studies (Sankaran and Holmes, 2019). It is biologically plausible that an individual's microbiome is comprised of numerous subcommunities, rather than just one, mixing together to varying degrees. The Latent Dirichlet Allocation (LDA) model describes such a generative process (Blei et al., 2003). Samples are defined by their mixture probabilities for each of the subcommunities rather than belonging to a single one. Technically speaking, LDA differs from the Dirichlet-multinomial mixture model by sampling the latent community variable repeatedly within a sample, once per sequencing read, rather than just once for the entire sample (Blei et al., 2003; Griffiths and Steyvers, 2004).

Latent Dirichlet Allocation has been successful in identifying functional subcommunities of the human gut and skin microbiota (Higashi et al., 2018; Sankaran and Holmes, 2019; Hosoda et al., 2020; Sommeria-Klein et al., 2020). Despite this, it has been noted that LDA is prone to over-smoothing of microbial counts, which are known to be sparse (Sankaran and Holmes, 2019). This can be attributed to the Dirichlet distribution being insufficient to capture the over-dispersion and zero-inflation of microbiome data. The distribution only has one dispersion parameter and inherently imposes a negative correlation between component counts, which may lead to spurious associations (Tang and Chen, 2019). Moreover, the model assumes that each species has a non-negative probability of belonging to every subcommunity. This implies that all species contribute to every subcommunity, even if only with low probability. Although, it is more likely that the presence of one species in a community prevents the presence of another.

As such, it would be advantageous to be able to identify community structures that are only composed of a subset of microbial species present in a data set. Thus, estimating some of the taxa membership probabilities for each subcommunity to be zero. We propose a zero-inflated Latent Dirichlet Allocation (zinLDA) model that is flexible enough to capture sparse subcommunities of microbiota. In the following section we detail the generative process of the LDA model and our zero-inflated LDA model. We also provide information on how to estimate model parameters using Markov chain Monte Carlo (MCMC) methods. We apply both models to simulation studies and real data analysis using data from the American Gut Project to directly compare the two and highlight how our proposed method provides better fit to microbiome data.

## 2. Materials and Methods

### 2.1. Notation and Terminology

Data in microbiome studies often comes from high-throughput sequencing of the 16S rRNA gene. A single biological sample can be represented by a vector of taxon counts with each component representing the number of reads aligned to that specific classification (e.g., ASV, species, genus). The following definitions and notations will be of help in defining a generative probabilistic model for microbiome studies:

- *w*_*dn*_ is the *nth* observed sequencing read in the *dth* biological sample. Sequencing reads are represented by *V*-length vectors with a single non-zero component whose value is equal to one, where *V* is the number of unique taxa in the study.
- $w_{dn}^{i}$ represents that the *nth* sequencing read in the *dth* sample belongs to the *ith* unique taxa (*i* = 1, …, *V*).
- ***w***_*d*_ = (*w*_*d*1_, …, *w*_*dN*_) is the *dth* biological sample consisting of *N* sequencing reads.
- A cohort **D** = (***w***_1_, …, ***w***_*D*_) is a collection of all biological samples in the study.

### 2.2. Latent Dirichlet Allocation (LDA)

Latent Dirichlet Allocation is a probabilistic model that is flexible enough to describe the generative process for discrete data in a variety of fields from text analysis to bioinformatics. When applied to microbiome studies, LDA provides the following generative process for the taxon counts in a cohort **D**:

1. For each of the *K* subcommunities, indexed by *j*:
  - Choose ***β***^(*j*)^ ~ Dir(η)
2. For each biological sample ***w*_*d*_** in the cohort:
  - Choose ***θ***^(*d*)^ ~ Dir(α)
3. For each of the *N* sequencing reads, *w*_*dn*_:
  - Choose a subcommunity, *z*_*dn*_ ~ Multinomial(1, ***θ***^(*d*)^)
  - Choose a taxon *w*_*dn*_ from *P*(*w*_*dn*_|*z*_*dn*_, ***β***), a multinomial probability distribution conditional on the subcommunity *z*_*dn*_.

**Figure 1:**
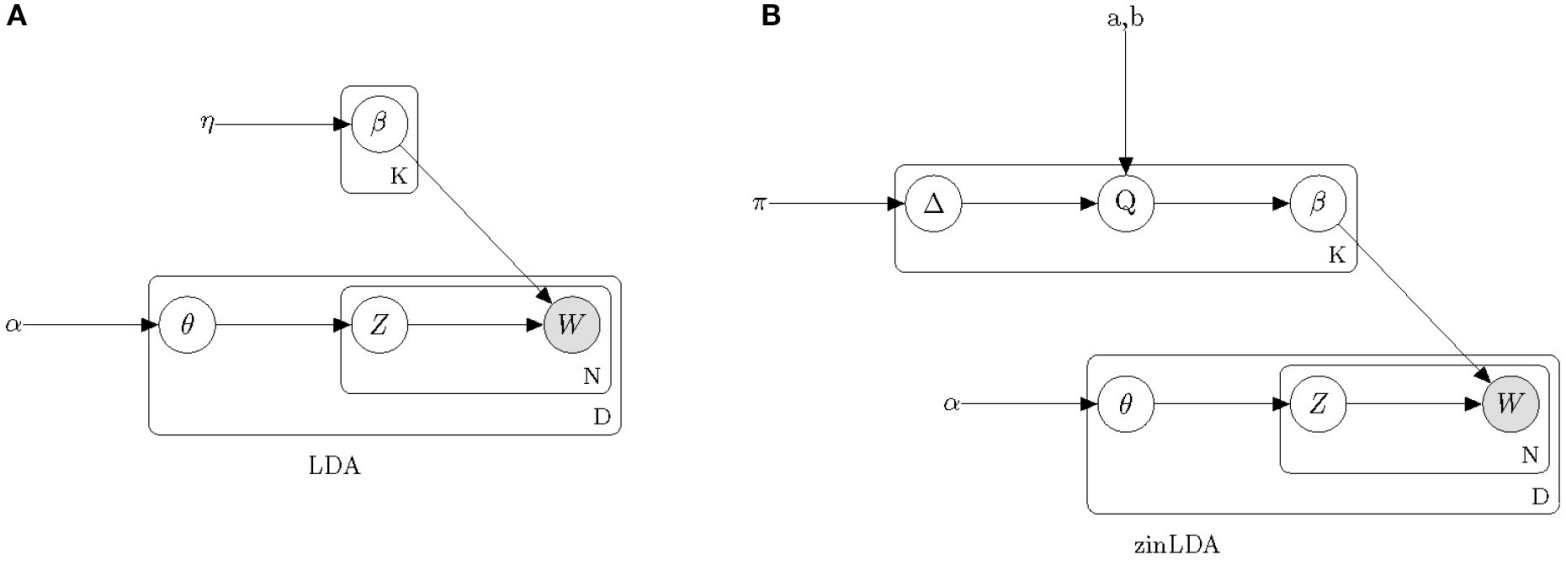
Plate diagrams of Latent Dirichlet Allocation **(A)** and zero-inflated Latent Dirichlet Allocation **(B)**. Nodes represent parameters and random variables, shading denotes the observed data. Boxes represent repeated sampling. The outer box is denoted with D for once per biological sample, the inner box with N for per sequencing read and the upper box with K for per subcommunity.

Figure 1 provides a graphical model representation of LDA. In this model, ***β*** = [β_*ij*_] fully describes the taxa distribution for each subcommunity. The probability that the *ith* taxa belongs to the *jth* subcommunity is denoted by β_*ij*_. Note that the taxa distribution is cohort-specific meaning that it is common across all samples and is only estimated once per cohort. The mixture probabilities for the subcommunities of the *dth* sample are denoted by a K-length vector, ***θ***^(*d*)^, with θ_*dj*_ representing the mixture probability of the *jth* subcommunity in the *dth* sample. Here, *K* is the number of underlying subcommunities and is assumed to be known a-priori. Additionally, *z*_*dn*_ is the subcommunity assignment for sequencing read *w*_*dn*_. Both hyperparameters η and α are assumed to be symmetric and are defined once for the whole cohort.

Intuitively, $\beta_{ij}=P\left(w_{dn}^{i}|z_{dn}=j\right)$ determines which taxa are important to subcommunity *j* and θ_*dj*_ = *P*(*z*_*dn*_ = *j*) determines which subcommunities are important in the *dth* sample. Moreover, the LDA model acts as a “soft” clustering technique by allowing samples to be composed of multiple subcommunities. Geometrically, the parameter space of ***β*** and ***θ*** can be thought of in terms of a simplex space. The taxa per subcommunity distribution belongs the *V*-1 simplex, such that ***β***^(*j*)^ ∈ *S*^*V*−1^. Meanwhile, ***θ***^(*d*)^, the subcommunity distribution per sample can be represented by a randomly selected point in the (*K*-1)-dimensional simplex, *S*^*K*−1^. This is different from the Dirichlet-Multinomial mixture model in which ***θ***^(*d*)^ = ***θ*** is assumed to be fixed across all samples and can be represented by the vertices of *S*^*K*−1^.

### 2.3. Zero-Inflated Latent Dirichlet Allocation (zinLDA)

We propose a modification to the Latent Dirichlet Allocation model that allows the latent subcommunity organization to be composed of both structural zeros, taxa that truly do not belong to the community, and sampling zeros, taxa that belong to the community, but are not captured due to low sequencing depth or dropout. Understanding and identifying the structural zeros in the data is biologically interesting as it provides insights into the absence of certain taxa in a given community.

The zero-inflated generalized Dirichlet (ZIGD) distribution is able to model both sources of zeros. The generalized Dirichlet (GD) distribution is an extension of the Dirichlet that allows for a more flexible covariance structure via the introduction of additional parameters (Connor et al., 1969). Though, it should be noted that the GD distribution alone does not model structural zeros.

To do so, we must modify the unique relationship between the GD distribution and a set of mutually independent beta random variables. By adding a zero-inflation probability, π, to each of the beta random variables we arrive at the zero-inflated generalized Dirichlet distribution. Formally, a length-*V* vector of ZIGD compositions, denoted by ***β*** = {β_1_, …β_*V*_}, can be formulated from a set of mutually independent zero-inflated beta random variables, which we denote by **Q** = {*Q*_1_, …, *Q*_*V*−1_}, with zero-inflation probabilities, ***π*** = {π_1_, …π_*V*−1_} and the parameters in the beta distributions denoted by (*a, b*). The relationship between the two random variables can be described as follows: β_1_ = *Q*_1_, $\beta_{i}=\prod_{l=1}^{i-1}\left(1-Q_{l}\right)$ for *l* = 2, …*V*−1, and $\beta_{V}=\overset{V-1}{\underset{i=1}{\sum }}\beta_{i}$ (Tang and Chen, 2019). Furthermore, we introduce an indicator variable, Δ_*i*_ = *I*(β_*i*_ = 0) = *I*(*Q*_*i*_ = 0), to identify structural zeros.

For every subcommunity *j*, let there be *L*_*j*_ taxa with β_*ij*_ > 0 ⇔ Δ_*ij*_ = 0. Then let **U**_*j*_ denote the set of indices of the non-zero taxa probabilities for subcommunity *j*, **U**_*j*_ = {*u*_1_*j*__, ..., *u*_*L*_*j*__}, and ***Ū***_*j*_ be its complement.

Replacing the Dirichlet(η) prior on ***β*** with a ZIGD(π, a, b) gives a zero-inflated Latent Dirichlet Allocation (zinLDA) model. The zinLDA model assumes the following generative process for a cohort **D**:

1. For each of the *K* subcommunities, indexed by *j*:
  - Choose **Δ**^(*j*)^ ~ Ber(π)
  - Choose ***β***^(*j*)^ ~ ZIGD(π, *a, b*)
2. For each biological sample ***w*_*d*_** in the cohort:
  - Choose ***θ*^(*d*)^** ~ Dir(α)
3. For each of the N sequencing reads, *w*_*dn*_:
  - Choose a subcommunity, *z*_*dn*_ ~ Multinomial(1, ***θ***^(*d*)^)
  - Choose a taxon, *w*_*dn*_ from *P*(*w*_*dn*_|*z*_*dn*_, ***β***), a multinomial probability distribution conditional on the subcommunity *z*_*dn*_.

In this model we assume hyperparameters π, *a, b*, and α are symmetric and are defined once for the whole cohort. Comparing the graphical model representation of zinLDA to that of the LDA model (Figure 1) underscores the differences between the two, particularly with respect to modeling ***β***.

We adopt a Bayesian framework for inference and parameter estimation. As such, inference for the zinLDA model is centered around the posterior distribution:

(1) $$\begin{array}{l} P\left(\theta ,\text{z},\beta ,\Delta |\text{w};\alpha ,\pi ,a,b\right)=\frac{P\left(\theta ,\text{z},\beta ,\Delta ,\text{w}|\alpha ,\pi ,a,b\right)}{P\left(\text{w}|\alpha ,\pi ,a,b\right)}. \end{array}$$

Calculation of this distribution cannot be done directly because the marginalization required to find the normalizing constant, *P*(**w**|α, π, *a, b*), is intractable. As such, approximate methods are necessary for parameter estimation. Variational inference may be used to find parameter estimates by maximizing an approximation to the true posterior. Alternatively, a Markov chain Monte Carlo procedure, such as Gibbs sampling, may be used to generate samples from the target posterior distribution for inference. It is worthy to note that due to the fact that both the Dirichlet and ZIGD distributions are conjugate prior for the multinomial distribution using a collapsed Gibbs sampler, marginalizing over ***β*** and ***θ***, gives a tractable solution, even more so than had collapsing not been performed. For this reason, we proposed a collapsed Gibbs sampler for the joint posterior distribution of **z** and **Δ** over taxa, *P*(**z**, **Δ**|**w**), where:

(2) $$\begin{array}{l} P\left(\text{z},\Delta |\text{w}\right)=\frac{P\left(\text{w, z},\Delta \right)}{P\left(\text{w}\right)}=\frac{P\left(\text{w}|\text{z},\Delta \right)P\left(\text{z}\right)P\left(\Delta |\pi \right)}{\sum_{\Delta }\sum_{z}P\left(\text{w, z},\Delta \right)} \end{array}$$

Integration over ***β*** and ***θ*** can be done separately as the former only appears in *P*(**w**|**z**, ***β***, **Δ**) and the latter only in *P*(**z**|***θ***). In Gibbs sampling, each state of the chain is taken as an assignment of each *z*_*dn*_ and Δ_*ij*_. These states are sampled conditional on the observed data and all the other parameters in the model at their current state. Thus, to perform the sampling, the full conditional distributions, *P*(*z*_*dn*_ = *j*|**w**, ***z***_−*n*_, **Δ**) and *P*(Δ_*ij*_ = 1|**w**, **z**, **Δ**_−*i*_), must be known. These distributions have closed form solutions due to the conjugate prior property of the Dirichlet and ZIGD distributions and can be found probabilistically (Supplementary Material):

(3) $$\begin{array}{l} P\left(z_{dn}=j|z_{-n},\text{w},\Delta \right)\\ \propto \left\{ \begin{array}{cc} \frac{a+n_{j,-n}^{\left(i\right)}}{a+n_{j,-n}^{\left(i\right)}+b_{ij}^{\left(z\right)}}\cdot \frac{m_{j,-n}^{\left(d\right)}+\alpha }{m_{.,-n}^{\left(d\right)}+K\alpha } & \text{if }i=u_{1_{j}}\\ \frac{a+n_{j,-n}^{\left(i\right)}}{a+n_{j,-n}^{\left(i\right)}+b_{ij}^{\left(z\right)}}\prod_{t<i,t\in U_{j}}\frac{b_{tj,-n}^{\left(z\right)}}{a+n_{j,-n}^{\left(t\right)}+b_{tj,-n}^{\left(z\right)}}\cdot \frac{m_{j,-n}^{\left(d\right)}+\alpha }{m_{.,-n}^{\left(d\right)}+K\alpha } & \text{if }u_{1_{j}}<i<u_{L_{j}}\\ \prod_{t<i,t\in U_{j}}\frac{b_{tj,-n}^{\left(z\right)}}{a+n_{j,-n}^{\left(t\right)}+b_{tj,-n}^{\left(z\right)}}\cdot \frac{m_{j,-n}^{\left(d\right)}+\alpha }{m_{.,-n}^{\left(d\right)}+K\alpha } & \text{if }i=u_{L_{j}}\\ 0 & \text{if }i\notin U_{j} \end{array}\right. \end{array}$$

(4) $$\begin{array}{l} P(\Delta_{ij}=1|\Delta_{-i},\text{w},\text{z})=\left\{ \begin{array}{cc} 0 & \text{if }n_{j}^{\left(i\right)}>0\\ \frac{\pi_{ij}}{\pi_{ij}+\left(1-\pi_{ij}\right)\frac{B\left(a_{ij}^{\left(z\right)},b_{ij}^{\left(z\right)}\right)}{B\left(a,b\right)}} & \text{if }n_{j}^{\left(i\right)}=0 \end{array}\right. \end{array}$$

where *z*_*dn*_ is the subcommunity assignment for sequencing read $w_{dn}^{i}$. We define $n_{j,-n}^{\left(i\right)}$ as the number of times the *ith* taxa is assigned to the *jth* subcommunity and $m_{j,-n}^{\left(d\right)}$ as the number of times the *jth* subcommunity occurs in the *dth* sample, both excluding the current subcommunity assignment of *z*_*dn*_. Additionally, we define $a_{ij}^{\left(z\right)}=a+n_{j}^{\left(i\right)}$ and $b_{ij}^{\left(z\right)}=b+n_{j}^{\left(i+1\right)}+\;...\;+n_{j}^{\left(V-1\right)}$.

The chain is initialized with informative values for the *z*_*dn*_ variables by sampling from a multinomial distribution with taxa probabilities equal to the β_*ij*_ estimates from a standard LDA model. Once the chain has been run long enough to guarantee sufficient convergence, a set of the initial runs is removed as a “burn-in” period, and the remaining are taken as a set of samples from the target posterior distribution. As such, for each run, we can calculate estimates of ***β*** and ***θ*** as follows using the posterior predictive distribution:

(5) $$\begin{array}{l} {\hat{\beta }}_{ij}=P\left(w_{new}^{\left(i\right)}|z_{new}^{\left(i\right)}=j,\text{w},\text{z},\Delta \right)\\ =\left\{ \begin{array}{cc} \frac{a+n_{j}^{\left(i\right)}}{a+n_{j}^{\left(i\right)}+b_{ij}^{\left(z\right)}} & \text{if }i=u_{1_{j}}\\ \frac{a+n_{j}^{\left(i\right)}}{a+n_{j}^{\left(i\right)}+b_{ij}^{\left(z\right)}}\prod_{t<i,t\in U_{j}}\frac{b_{tj}^{\left(z\right)}}{a+n_{j}^{\left(t\right)}+b_{tj}^{\left(z\right)}} & \text{if }u_{1_{j}}<i<u_{L_{j}}\\ \prod_{t<i,t\in U_{j}}\frac{b_{tj}^{\left(z\right)}}{a+n_{j}^{\left(t\right)}+b_{tj}^{\left(z\right)}} & \text{if }i=u_{L_{j}}\\ 0 & \text{if }i\notin U_{j} \end{array}\right. \end{array}$$

(6) $$\begin{array}{l} {\hat{\theta }}_{j}^{\left(d\right)}=P\left(z_{new}=j|\text{z}\right)=\frac{m_{j}^{\left(d\right)}+\alpha }{m_{.}^{\left(d\right)}+K\alpha } \end{array}$$

The final estimate of ***θ*** is defined as its posterior mean across all the runs. The final estimate of ***β*** can be found in a two-part process. First, calculate the posterior mean of Δ_*ij*_ across all runs, which is equivalent to a posterior estimate of π_*ij*_. Then dichotomize ${\hat{\pi }}_{ij}$ according to $I\left({\hat{\pi }}_{ij}\geq 0.5\right)$. Next, assign ${\hat{\beta }}_{ij}=0$ for any dichotomized ${\hat{\pi }}_{ij}=1$, otherwise assign ${\hat{\beta }}_{ij}$ its respective posterior mean and normalize within each subcommunity such that $\underset{i}{\sum }\beta_{ij}=1$.

## 3. Results

### 3.1. Simulation Study

We conducted a simulation study to compare estimation accuracy and model fit between the proposed zinLDA and the standard LDA models. The data was simulated from a true zinLDA model, following the steps specified by the generative algorithm given section 2.3. First, we selected the total number of taxa (*V*) to be 120 across 150 independent microbial samples. Next, the total number of reads in each sample were drawn from a discrete uniform distribution with a lower bound of 5,000 and upper bound of 25,000. These parameters were selected to reflect real microbiome data sets aggregated to the genus-level classification. The number of subcommunities (*K*) was selected as five. The hyperparameter α of the Dirichlet distribution on ***θ*** was set to 50/*K*, as suggested for the original LDA model (Griffiths and Steyvers, 2004). Additionally, the hyperparameters π, *a*, and *b* of the zero-inflated generalized Dirichlet distribution on ***β*** were set to 0.4, 0.05, and 10, respectively. After running the simulation algorithm, the taxa that had a zero count for every sample, meaning a prevalence of 0%, were removed as such taxa would not be observed in a real data analysis. This reduced the total number of observed taxa (*V*_*obs*_) to 87.

A zinLDA model with five subcommunities was fit to the simulated data set. Hyperparameters α, π, *a*, and *b* were set to their true values, as specified under simulation. Likewise, a standard LDA model with five subcommunities was fit, with default hyperparameter values of 50/*K* and 0.1 for α and η, respectively (Griffiths and Steyvers, 2004). To deal with the label switching problem commonly seen in Bayesian inference with mixture models, we use a method previously proposed to compare labels from an LDA model to their ground-truth. The pairwise Pearson correlation was calculated for each true-estimated subcommunity pair. The pair with the highest correlation is matched, then the pair with the next highest correlation among the remaining is matched, and so on until all true-estimated pairs are uniquely matched (Sankaran and Holmes, 2019).

**Figure 2:**
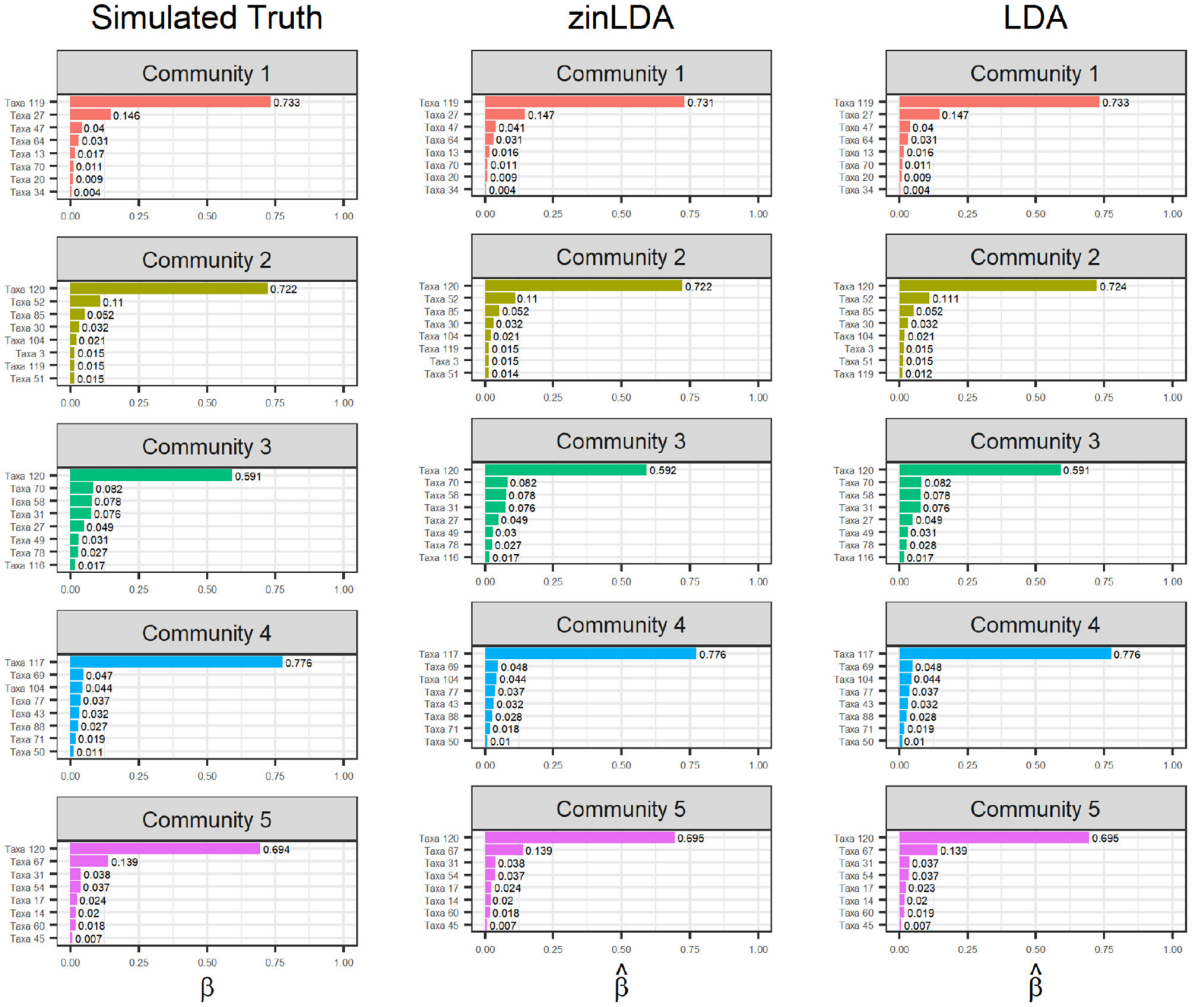
Bar graphs of the top eight taxa, for each of the five sub-communities, with their corresponding β_*ij*_ values. The first column contains the “ground truth” top taxa from simulation. The second and third columns are the estimated top taxa from the zinLDA and LDA models, respectively.

To determine how well zinLDA is able to capture the latent community structure we compare the estimated β_*ij*_ for the top eight taxa per community to their true value and estimated value from the standard LDA model. Figure 2 shows that both zinLDA and LDA correctly identify all of the top microbial taxa for each of the five subcommunities. Moreover, estimates from both models show low bias. We investigated how misspecification of the number of subcommunities influences zinLDA's ability to recover the representative taxa. An under-specified model, with one too few communities, collapses the representative taxa of two of the subcommunities together. Thus, resulting in both upwardly and downwardly biased estimates of β_*ij*_, the taxa over subcommunity probabilities. The remaining three subcommunities have their representative taxa recovered and their respective β_*ij*_ estimates were not affected. Likewise, for an over-specified model, with one too many communities, it is able to accurately detect the five true subcommunity structures as specified under simulation, but identifies an additional nonsensical subcommunity that is composed of only one taxa (Supplementary Figure 1).

**Figure 3:**
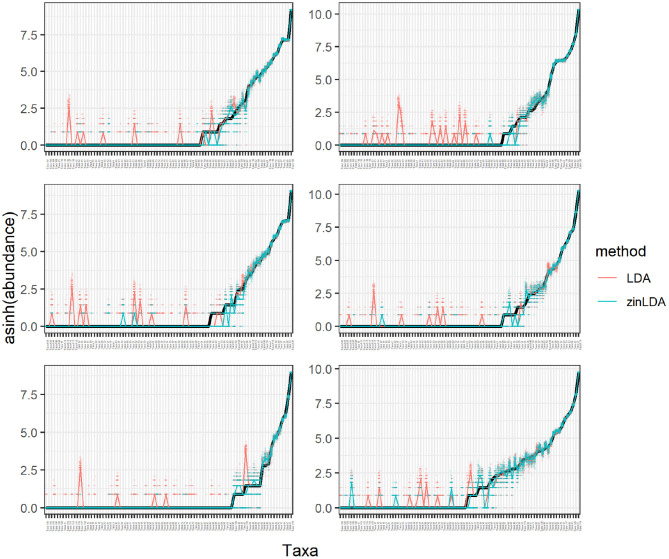
Observed and posterior predictive simulated *asinh*-transformed taxon counts plotted in order of increasing true abundance. Each panel is a different biological sample. The solid black line represents the true counts. The pink and blue points are the counts from 50 posterior predictive simulated data sets and the pink and blue solid line represents the median counts across all 50 data sets, from the LDA and zinLDA models, respectively.

Fit of the two models was assessed through posterior predictive checks (Gelman et al., 1996). For each model, the posterior predictive distribution was used to simulate 100 data sets of the same dimensions as the original. The rationale behind using posterior predictive checks to assess model fit is as follows: if the model provides reasonable fit then the data simulated from the posterior predictive distribution, which is conditional on the observed data (*X*_*obs*_) and the current model, should “look similar” to the observed data. We quantify how similar the observed data and the posterior predictive simulated data are by the test statistic *T*(*X*) = *X*_*i*·_, the count for the *ith* taxa. Figure 3 plots the results from the posterior predictive checks. Each panel corresponds to a single biological sample. The *y*-axis plots *T*(*X*) on the *asinh* scale. The *x*-axis plots each of the 87 taxa, ordered from smallest to largest based on the observed data for that sample. For large taxon counts we see that both models do well, with median values of both being similar to the truth, or observed, values. In contrast, we see that for small taxon counts the zinLDA model outperforms LDA. Specifically, for zero counts the zinLDA model is able to accurately estimate these counts better than its LDA counterpart. Across the 50 data sets simulated from the posterior predictive distribution, the zinLDA exhibits less over-smoothing for small taxon counts compared to the original LDA. Thus, this is an indication that the zinLDA model provides better fit to the data than the LDA.

To quantify how well the zinLDA model is able to distinguish between rare and absent taxa in each subcommunity we calculate the sensitivity, specificity, positive predictive value (PPV), and negative predictive value (NPV). We define a “positive” outcome as being a structural zero, Δ_*ij*_ = 1, and a “negative” outcome as being a non-zero probability of belonging to that subcommunity, Δ_*ij*_ = 0. The results show that under these simulation settings zinLDA can differentiate sampling and structural zeros with reasonable sensitivity and specificity (Table 1). Upon further examining the data, we noticed that the model we used to generate the data resulted in many taxa with very small true non-zero probabilities, making it very difficult to separate sampling zeros from structural zeros. To further demonstrate this point, we ran two additional simulations to see how different model parameters affect the posterior inference of being structural zeros. Both simulations reduce the number of taxa (*V*) to 50, but one also changes hyperparameter *a*, of the ZIGD distribution, to 0.5 from 0.05. Table 1 shows that reducing the number of taxa without changing the value of *a* reduces the model's ability to differentiate between the two sources of zeros. In contrast, reducing *V* and also increasing *a* significantly increases the model's ability to accurately detect structural zeros, with such a modeling having sensitivity of 0.9 and PPV of 0.92. The sharp difference in the values of these diagnostic metrics between the models can be attributed to the fact that *V*, *a*, and *b* all influence the β_*ij*_ values, which in turn influences the probability of observing a sampling zero. For example, decreasing *V* without changing *a* reduces many of the β_*ij*_ values, thus increasing the probability of observing a sampling zero. On the other hand, decreasing *V* in conjunction with increasing *a* increases many of the β_*ij*_ values and therefore decreases the probability of observing a sampling zero.

**Table 1:** Comparison of estimated structural zero taxa from the zinLDA model to true structural zero taxa from simulation across different parameter settings using sensitivity, specificity, positive predictive value, and negative predictive value.

	**Sensitivity**	**Specificity**	**PPV**	**NPV**
*V* = 50, *a* = 0.5	0.90	0.94	0.92	0.93
*V* = 50, *a* = 0.05	0.51	0.51	0.40	0.61
*V* = 87, *a* = 0.05	0.73	0.67	0.59	0.79

*A “positive” results is assumed to be β_ij_ = 0*.

### 3.2. Real Data Applications

The American Gut Project (AGP) is a self-selected and open platform cohort. Citizen-scientists primarily in the United States, United Kingdom, and Australia, opted into the study, paid a fee to offset the cost of sample processing and sequencing, and gave informed consent (McDonald et al., 2018). All subjects provided a fecal microbiome sample and self-reported meta-data. The sequencing protocol used was identical to that of the Earth Microbiome Project (Gilbert et al., 2014; McDonald et al., 2018). The AGP microbial 16S rRNA gene sequencing data and meta-data are publicly available in The European Bioinformatics Institute repository under the accession ERP012803.

This analysis used a prior subset of the AGP data consisting of 3,679 subjects. Reads that were ambiguously assigned or unassigned at the genera level were removed. Moreover, genera with a prevalence of <20% across all samples were removed. After this filtering of the microbial genera, any samples with a total number of reads of zero were removed. This left 3,566 samples and 70 unique genera for downstream analyses.

A random subset of 1,000 subjects from the AGP data was sampled, a zinLDA model with five subcommunities and hyperparameter values being specified the same way as in the simulation study was fit. When possible, the choice of the number of latent subcommunities should be informed by biological or clinical reasoning. In the absence of such, data-driven approaches may be used. In particular for the AGP data, *K* was determined by comparing the log-likelihood, AIC, and representative taxa across many models, each with a different number of subcommunities, applied to a set of 1,000 independently selected subjects. These results were robust across slight changes in the number of subcommunities.

**Figure 4:**
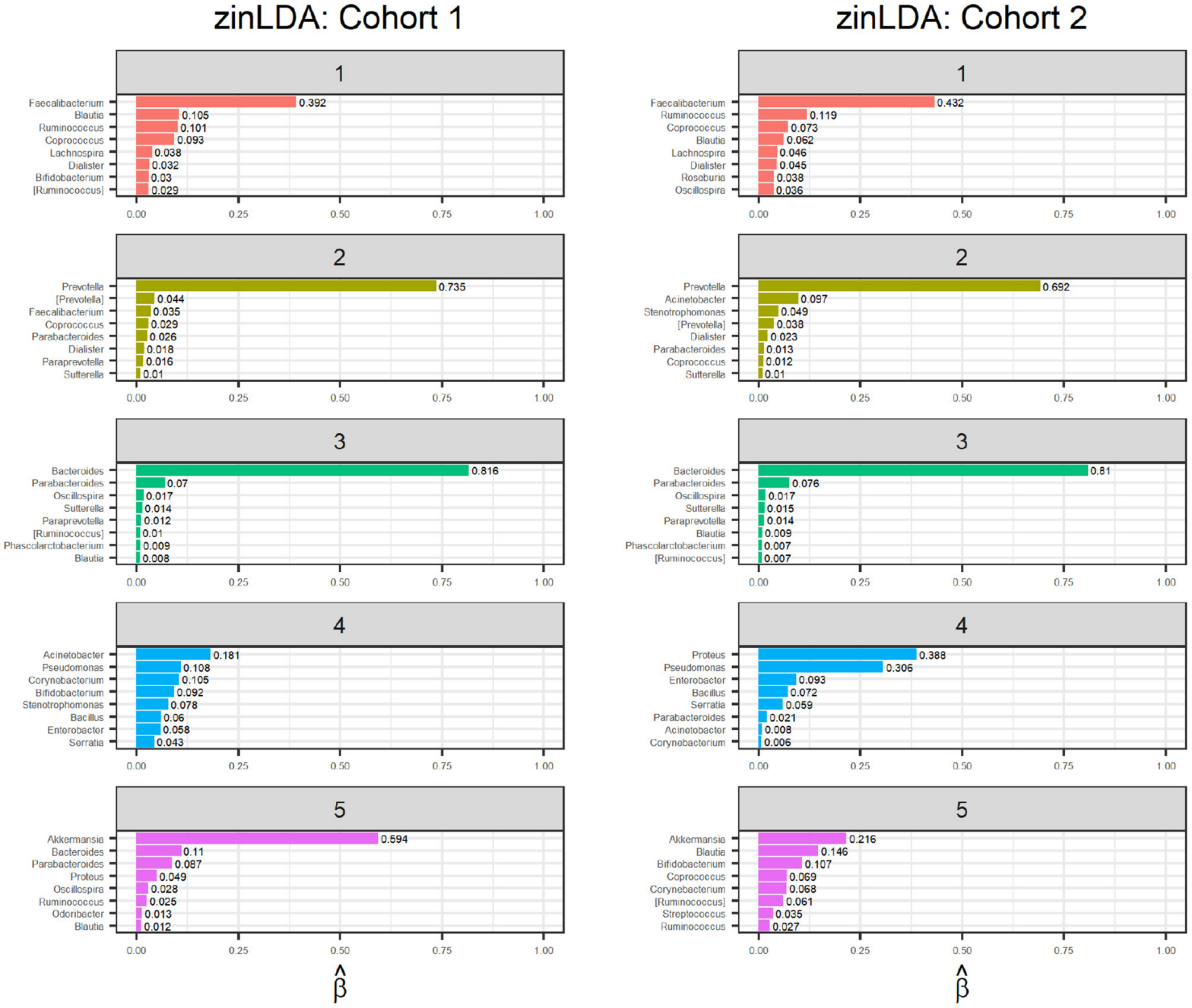
Bar graphs of the representative taxa for each of the five sub-communities, with their corresponding β_*ij*_ values, for two independent subsets of 1,000 randomly selected subjects from the American Gut Project.

The representative taxa from each subcommunity and their membership probability (β_*ij*_) is shown in Figure 4. We observe that each subcommunity is characterized by one single dominant taxa, including *Faecalibacteruim, Prevotella, Bacteroides, Acinetobacter*, and *Akkermansia*.

Model fit was assessed via posterior predictive checks and compared to that of the standard LDA model. Since current sequencing technology, such as 16S rRNA gene sequencing, can only provide quantification about relative abundance model fit was assessed using both the relative abundance and the observed counts (Supplementary Figures 2, 3). The two plots exhibit similar patterns, indicating the difficulty in fitting the small count data. Another explanation of observing such similar model fits is that our analysis did not identify structural zeros with strong evidence in our data. Supplementary Figure 4 shows the posterior estimates of the probability of zero count being a structural zero for each of the taxa in each subcommunity, indicating relatively weak evidence of being structural zeros.

Finally, to determine whether the model is stable, meaning it detects true subcommunity clusters of co-occurring taxa and is not clustering the noise in the observations, we apply an identical zinLDA model to another set of 1,000 AGP microbial samples that is independent from the first. The representative taxa from this validation set is compared to that of the first cohort (Figure 4). The subcommunities between the two cohorts were matched using pairwise correlations as done in simulations. The average cosine similarity of the matched subcommunities is 0.80. The results show that the communities identified by zinLAD are very stable and replicable.

## 4. Discussion

The micro-organisms that constitute the human microbiome form subcommunity-like structures via dynamic and complex interactions with one another. Identifying these structures is imperative for a better understanding of how these microbes influence human-host health. We propose a zero-inflated latent Dirichlet allocation model, a further modification of the LDA model that amounts to changing the prior distribution on the taxa per subcommunity distribution to a zero-inflated generalized Dirichlet from a Dirichlet distribution. Despite this change our model retains the advantageous conjugate prior property between the ZIGD and multinomial distributions. As such, we are able to implement an efficient Gibbs sampling algorithm, with only one additional step compared to that of LDA, for parameter estimation.

zinLDA modifies the LDA model proposed by Blei et al. (2003) to allow for subcommunities to be composed of a subset of all the microbes in a cohort of samples. Mathematically, since a subcommunity is defined as a distribution over taxa, this is equivalent to assigning some taxa a zero-probability of belonging to it. This is particularly advantageous in microbial analyses as it allows for a clear distinction between sampling and structural zeros within a subcommunity structure. Structural zeros come from those zero-probability taxa; they are truly absent from the community. Sampling zeros come from taxa that do belong to the community, but with low probability, and thus were not captured due to shallow sequencing depth. Due to this adjustment, zinLDA model can be used to simulate more realistic sparse count data than models such as the Dirichlet multinomial or Dirichlet multinomial mixture models.

We used simulation studies to compare the two models and investigate where zinLDA outperforms the standard LDA model. First, we show that the two performed equally well in identifying the representative taxa for each subcommunity. This is to be expected as the LDA model already does a good job in identifying common taxa and the zinLDA estimates of the community assignment for each sequencing read were initialized using the results from a standard LDA model. The performance gain in using zinLDA is seen when examining the low probability and absent taxa within in each subcommunity. The greatest performance gains are made when the probability of being a sampling zero is not too small. Furthermore, we use real data from the citizen scientists of the American Gut Project to show that our method can detect potentially meaningful biological and ecological subcommunities of microbial species. By assigning each sample a probability of belonging to each of these subcommunities we are also able to gather information about population level microbial structures.

As for any Bayesian models, zinLDA requires the hyperparameters to be pre-specified. In our analysis of the real data sets, we used the same hyperparameters as in our simulations and explored various other choices. For the same number of communities, we observed that the community structures and the representative taxa were not too sensitive to the values of these hyperparameters. Determining the number of clusters or subcommunities is a hard problem, as for any clustering methods. For real data analysis, we suggest that the users try different numbers of *K*, evaluate the sub-community structures, and then choose one based on both the sizes of the communities and also possible biological interpretations.

Finally, the zinLDA model can be used to simulate more realistic microbiome count data that allow for both structural zeros and sampling zeros. Such simulations can be used to evaluate various statistical tests developed for microbiome data analysis, including evaluating power of the tests for differential abundance and methods for modeling microbiome count data.

## Data Availability Statement

Publicly available datasets were analyzed in this study. The datasets for this study can be found in EBI under project PRJEB11419 and Qiita study ID 10317. Software for implementing the method described in this manuscript is publicly available on GitHub at https://github.com/rebeccadeek/zinLDA.

## Author Contributions

RD and HL developed the ideas and the methods together, analyzed the real data sets, and wrote the manuscript. RD implemented the methods and performed the numerical analysis.

## Conflict of Interest

The authors declare that the research was conducted in the absence of any commercial or financial relationships that could be construed as a potential conflict of interest.
