# Supplementary material for: A Zero-Inflated Latent Dirichlet Allocation Model for Microbiome Studies
Source: Front Genet. 2021 Jan 22;11:602594. doi: 10.3389/fgene.2020.602594 (PMC7862749; doi:10.3389/fgene.2020.602594)
Supplement: Supplementary file 1 [file Data_Sheet_1.PDF]

Supplementary Material

1 SUPPLEMENTARY DATA

1.1 Notation and Terminology

The following defines the parameters, and their notation, of the proposed zero-inflated Latent Dirichlet Allocation model:

1.1.1 Parameters

- $\boldsymbol{z}^{(d)} = (z_{d1}, \ldots, z_{dN})$: is the vector of subcommunity assignments for the dth biological sample. $z_{dn} = j$ indicates the nth sequencing read in the dth sample belongs to the jth subcommunity. There are K underlying/latent subcommunity variables.
- $\boldsymbol{\theta}^{(d)} = (\theta_{d1}, \ldots, \theta_{dK})$: is the vector of mixture probabilities for the dth biological sample. $\theta_{dj} = P(z = j|\boldsymbol{\theta}^{(d)})$ is the mixture probability for the jth subcommunity in the sample.
- $\boldsymbol{\beta}^{(j)} = (\beta_{1j}, \ldots \beta_{(V-1)j})$ and $\beta_{Vj} = 1 - \sum_{i=1}^{V-1} \beta_{ij}$: is the vector of taxon probabilities for the jth subcommunity. $\beta_{ij} = P(w^i|z = j, \boldsymbol{\beta})$, the probability of observing the ith taxon under the jth subcommunity
- $\boldsymbol{Q}^{(j)} = (Q_{1j}, \ldots, Q_{(V-1)j})$: the set of independent zero-inflated Beta random variables used to construct $\boldsymbol{\beta}^{(j)}$
- $\boldsymbol{\alpha}^{(d)} = (\alpha_{d1}, \ldots, \alpha_{dK})$: hyperparameter of the Dirichlet prior of $\boldsymbol{\theta}^{(d)}$
- $\boldsymbol{\pi}^{(j)} = (\pi_{1j}, \ldots, \pi_{(V-1)j})$: hyperparameter of the ZIGD specifying the probability of being a structural zero for the ith taxon under the jth subcommunity
- $\boldsymbol{\Delta}^{(j)} = (\Delta_{1j}, \ldots, \Delta_{(V-1)j})$: is the vector of indicator variables, where $\Delta_{ij} = I(Q_{ij} = 0) = I(\beta_{ij} = 0)$ is an indicator function for structural zeros
- $\mathbf{a}^{(j)} = (a_{1j}, ..., a_{(V-1)j})$: hyperparameter on the ZIGD of $\boldsymbol{\beta}^{(j)}$
- $\mathbf{b}^{(j)} = (b_{1j}, ..., b_{(V-1)j})$: hyperparameter on the ZIGD of $\boldsymbol{\beta}^{(j)}$

Note that in all subsequent derivations the hyperparameters $\boldsymbol{\alpha}^{(d)}, \boldsymbol{\pi}^{(j)}, \mathbf{a}^{(j)}, \mathbf{b}^{(j)}$ are all assumed to be symmetric such that, $\alpha_{dj} = \alpha, \pi_{ij} = \pi, a_{ij} = a,$ and $b_{ij} = b \ \forall i, j$.

1.2 Model Derivation and Inference

1.2.1 Probability Model

The zero-inflated Latent Dirichlet Allocation model is a completely probabilistic hierarchical model with the following specification:

$$\begin{aligned} w_{dn}|z_{dn}, \boldsymbol{\beta}^{(z_{dn})} &\sim \text{Multinomial}(\boldsymbol{\beta}^{(z_{dn})}) \\ \boldsymbol{\beta}^{(z_{dn})}|\boldsymbol{\Delta} &\sim \begin{cases} \text{I}(\beta_{i,z_{dn}} = 0) & \text{if } \Delta_{i,z_{dn}} = 1 \\ \text{GD}(a, b) & \text{if } \Delta_{i,z_{dn}} = 0 \end{cases} \\ \boldsymbol{\Delta}|\pi &\sim \text{Ber}(\pi) \\ z_{dn}|\boldsymbol{\theta}^{(d)} &\sim \text{Multinomial}(\boldsymbol{\theta}^{(d)}) \\ \boldsymbol{\theta}^{(d)} &\sim \text{Dirichlet}(\alpha) \end{aligned}$$

1.2.2 Posterior Distribution

Such a hierarchical model readily lends itself to the Bayesian framework of inference, where the key inferential distribution is the posterior distribution. In zinLDA the posterior is the joint distribution of the four latent variables, given the observed sequencing reads: $P(\boldsymbol{\theta}, \mathbf{z}, \boldsymbol{\beta}, \boldsymbol{\Delta}|\mathbf{w}; \alpha, \pi, a, b)$. To find an analytical form of the posterior we must first find the joint distribution, $P(\boldsymbol{\theta}, \mathbf{z}, \boldsymbol{\beta}, \boldsymbol{\Delta}, \mathbf{w}|\alpha, \pi, a, b)$, and then marginalize to find the normalizing constant, $P(\mathbf{w}|\alpha, \pi, a, b)$. The joint distribution can be found as follows:

$$\begin{aligned} P(\boldsymbol{\theta}, \mathbf{z}, \boldsymbol{\beta}, \boldsymbol{\Delta}, \mathbf{w}|\alpha, \pi, a, b) &= P(\mathbf{w}|\mathbf{z}, \boldsymbol{\beta}) P(\boldsymbol{\theta}, \mathbf{z}, \boldsymbol{\beta}, \boldsymbol{\Delta}|\alpha, \pi, a, b) \\ &= \prod_{d=1}^{D} P(\boldsymbol{\theta}^{(d)}|\alpha) P(\boldsymbol{\beta}|\boldsymbol{\Delta}, a, b) P(\boldsymbol{\Delta}|\pi) \prod_{n=1}^{N} P(w_{dn}|z_{dn}, \boldsymbol{\beta}) P(z_{dn}|\boldsymbol{\theta}^{(d)}) \\ &= \prod_{d=1}^{D} \text{Dir}(\alpha) P(\boldsymbol{\beta}|\boldsymbol{\Delta}, a, b) \text{Ber}(\pi) \prod_{n=1}^{N} \prod_{i=1}^{V} (\beta_{ij}\theta_j)^{w_{dn}^i} \end{aligned}$$

$$\begin{aligned} \text{Where: } \boldsymbol{\beta}^{(j)}|\boldsymbol{\Delta}^{(j)}, a, b &\sim \begin{cases} \text{I}(\beta_{ij} = 0) & \text{if } \Delta_{ij} = 1 \\ \text{GD}(a, b) & \text{if } \Delta_{ij} = 0 \end{cases} \\ P(\boldsymbol{\beta}^{(j)}|\boldsymbol{\Delta}^{(j)}, a, b) &= \text{I}(\boldsymbol{\beta}_{\bar{U}_j} = 0) \text{GD}(\mathbf{a}_{U_j}, \mathbf{b}_{U_j}) \\ P(\boldsymbol{\beta}|\boldsymbol{\Delta}, a, b) P(\boldsymbol{\Delta}|\pi) = P(\boldsymbol{\beta}, \boldsymbol{\Delta}|\pi, a, b) &= P(\boldsymbol{\beta}|\pi, a, b) \sim \text{ZIGD}(\pi, a, b) \end{aligned}$$

It should be noted that the probability equality statement: $P(\boldsymbol{\beta}, \boldsymbol{\Delta}|\pi, a, b) = P(\boldsymbol{\beta}|\pi, a, b)$ holds since the addition of $\boldsymbol{\Delta}$ does not give any additional information about $\boldsymbol{\beta}$. Meaning that $\beta_{ij} = 0$ if and only if $\Delta_{ij} = 1$. Moreover, here w_{dn}^i is an indicator that the nth sequencing read in the dth document corresponds to the ith unique taxon.

The Dirichlet density is given by:

$$Dir(\alpha) = \frac{\Gamma\left(\sum_{j=1}^{K} \alpha\right)}{\prod_{j=1}^{K} \Gamma(\alpha)} \prod_{j=1}^{K} \theta_{dj}^{\alpha-1}$$

The GD density is given by:

$$GD(a,b) = \prod_{i=1}^{V-1} \frac{\Gamma(a+b)}{\Gamma(a)\Gamma(b)} \beta_{ij}^{(a-1)} (1 - \beta_{1j} - ... - \beta_{ij})^{c_{ij}}$$

Where $c_{ij} = b_{ij} - a_{(i+1)j} - b_{(i+1)j} = -a$ for $i = 1, ..., V-2$ and $c_{(V-1)j} = b_{(V-1)j} - 1 = b - 1$ under the assumption of symmetry of a and b

The Bernoulli density is given by:

$$Ber(\pi) = \prod_{j=1}^{K} \prod_{i=1}^{V-1} \pi^{\Delta_{ij}} (1-\pi)^{1-\Delta_{ij}}$$

There is no closed-form expression for this posterior due to the marginalization required to find the normalizing constant. As such we propose a Gibbs sampler for the collapsed posterior distribution $P(\mathbf{z}, \mathbf{\Delta}|\mathbf{w})$:

$$P(\mathbf{z}, \mathbf{\Delta}|\mathbf{w}) = \frac{P(\mathbf{w}|\mathbf{z}, \mathbf{\Delta}) P(\mathbf{z}) P(\mathbf{\Delta}|\pi)}{\sum_{\Delta} \sum_{z} P(\mathbf{w}, \mathbf{z}, \mathbf{\Delta})}$$

Since $\boldsymbol{\beta}$ and $\boldsymbol{\theta}$ only appear in $P(\mathbf{w}|\mathbf{z}, \boldsymbol{\beta}, \mathbf{\Delta})$ and $P(\mathbf{z}|\boldsymbol{\theta})$, respectively, the integration required to marginalize over the two can be done separately. Due to the conjugate prior property of both the zero-inflated generalized Dirichlet and the Dirichlet, the marginalization results in known compound probability distributions. We begin by marginalizing over $\boldsymbol{\beta}$:

$$
\begin{aligned}
P(\mathbf{w}|\mathbf{z},\boldsymbol{\Delta}) &= \int P(\mathbf{w}|\mathbf{z},\boldsymbol{\beta})P(\boldsymbol{\beta}|\boldsymbol{\Delta})d\boldsymbol{\beta} \\
&= \prod_{j=1}^{K}\int \text{Multinomial}(\boldsymbol{\beta}^{(j)})I(\boldsymbol{\beta}_{\bar{U}_j}=\mathbf{0})\text{GD}(\boldsymbol{a}_{U_j},\boldsymbol{b}_{U_j})d\boldsymbol{\beta}^{(j)} \\
&= \prod_{j=1}^{K}\int \prod_{i=1}^{V}\beta_{ij}^{n_j^{(i)}}\prod_{l=1_j}^{L_j-1}\frac{1}{B(a,b)}\beta_{u_lj}^{a-1}(1-\beta_{u_{1_j}j}-\cdots-\beta_{u_lj})^{c_{u_l j}}d\boldsymbol{\beta}^{(j)} \\
&= \prod_{j=1}^{K}\int \prod_{l=1_j}^{L_j-1}\beta_{u_lj}^{n_j^{(u_l)}}\frac{1}{B(a,b)}\beta_{u_lj}^{a-1}(1-\beta_{u_{1_j}j}-\cdots-\beta_{u_lj})^{c_{u_l j}}d\boldsymbol{\beta}^{(j)} \\
&= \prod_{j=1}^{K}\prod_{i\in U_j}\frac{B(a_{ij}^{(z)},b_{ij}^{(z)})}{B(a,b)} = \prod_{j=1}^{K}\prod_{i\in U_j}\frac{\Gamma(a_{ij}^{(z)})\Gamma(b_{ij}^{(z)})}{\Gamma(a_{ij}^{(z)}+b_{ij}^{(z)})}\frac{\Gamma(a+b)}{\Gamma(a)\Gamma(b)} \\
&= \prod_{j=1}^{K}\frac{B(\mathbf{a}_{U_j}^{(z)},\mathbf{b}_{U_j}^{(z)})}{B(\mathbf{a}_{U_j},\mathbf{b}_{U_j})}
\end{aligned}
$$

Where we define $n_j^{(i)}$ as the number of times the ith taxa is assigned to the jth subcommunity, $a_{u_lj}^{(z)} = a + n_j^{(u_l)}$, and $b_{u_lj}^{(z)} = b + n_j^{(u_l+1)} + \cdots + n_j^{(u_{L_j})}$. The ratio of the two beta functions is defined to be one for the last element of each U_j.

Likewise, we marginalize over $\boldsymbol{\theta}$:

$$
\begin{aligned}
P(\mathbf{z}) &= \int P(\mathbf{z}|\boldsymbol{\theta})P(\boldsymbol{\theta})d\boldsymbol{\theta} \\
&= \prod_{d=1}^{D}\int \text{Multinomial}(\boldsymbol{\theta}^{(d)})\text{Dir}(\alpha)d\boldsymbol{\theta}^{(d)} \\
&= \prod_{d=1}^{D}\int \left(\prod_{j=1}^{K}\theta_{dj}^{m_j^{(d)}}\right)\frac{\Gamma(\sum_{j=1}^{K}\alpha)}{\prod_{j=1}^{K}\Gamma(\alpha)}\prod_{j=1}^{K}\theta_{dj}^{\alpha-1}d\boldsymbol{\theta}^{(d)} \\
&= \prod_{d=1}^{D}\frac{\Gamma(K\alpha)}{\Gamma(\alpha)^K}\frac{\prod_{j=1}^{K}\Gamma(m_j^{(d)}+\alpha)}{\Gamma(\sum_{j=1}^{K}m_j^{(d)}+\alpha)} \\
&= \left(\frac{\Gamma(K\alpha)}{\Gamma(\alpha)^K}\right)^D\prod_{d=1}^{D}\frac{\prod_{j=1}^{K}\Gamma(m_j^{(d)}+\alpha)}{\Gamma(m_\cdot^{(d)}+K\alpha)}
\end{aligned}
$$

Where we define $m_j^{(d)}$ as the number of times the jth subcommunity occurs in the dth biological sample.

1.3 Gibbs Sampling of $P(\mathbf{z}, \boldsymbol{\Delta}|\mathbf{w})$

Since the posterior distribution $P(\mathbf{z}, \boldsymbol{\Delta}|\mathbf{w})$ cannot be directly computed we use Gibbs sampling to sequentially sample each z_{dn} and Δ_{ij} individually conditional on all other $\mathbf{z}$ and $\boldsymbol{\Delta}$. These samples are taken as draws from the target posterior distribution which can be used to approximate inferential quantities of interest. To do so we find the full conditional distributions:

1. $P(z_{dn} = j|\mathbf{w}, \boldsymbol{z}_{-n}, \boldsymbol{\Delta})$
2. $P(\Delta_{ij} = 1|\mathbf{w}, \mathbf{z}, \boldsymbol{\Delta}_{-i})$

1.3.1 Gibbs Sampling of $P(z_{dn} = j|\mathbf{w}, \boldsymbol{\Delta}, \boldsymbol{z}_{-i})$

$$
\begin{aligned}
P(z_{dn}^{(i)} = j|\boldsymbol{z}_{-n}, \mathbf{w}, \Delta) &= \frac{P(z_{dn}^{(i)} = j, \boldsymbol{z}_{-n}, \mathbf{w}, \Delta)}{P(\boldsymbol{z}_{-n}, \mathbf{w}, \Delta)} = \frac{P(\mathbf{z}, \mathbf{w}, \Delta)}{P(\boldsymbol{z}_{-n}, \mathbf{w}, \Delta)} \\
&= \frac{P(\mathbf{w}|\mathbf{z}, \boldsymbol{\Delta})P(\mathbf{z})P(\boldsymbol{\Delta}|\pi)}{P(\mathbf{w}|\boldsymbol{z}_{-n}, \boldsymbol{\Delta})P(\boldsymbol{z}_{-n})P(\boldsymbol{\Delta}|\pi)} \\
&= \frac{P(\mathbf{w}|\mathbf{z}, \boldsymbol{\Delta})P(\mathbf{z})}{P(\mathbf{w}|\boldsymbol{z}_{-n}, \boldsymbol{\Delta})P(\boldsymbol{z}_{-n})}
\end{aligned}
$$

$$
P(z_{dn}^{(i)} = j|\boldsymbol{z}_{-n}, \mathbf{w}, \Delta) \propto \begin{cases}
\frac{a+n_{j,-n}^{(i)}}{a+n_{j,-n}^{(i)}+b_{ij}^{(z)}} \cdot \frac{m_{j,-n}^{(d)}+\alpha}{m_{.,-n}^{(d)}+K\alpha} & \text{if } i = u_{1_j} \\
\frac{a+n_{j,-n}^{(i)}}{a+n_{j,-n}^{(i)}+b_{ij}^{(z)}} \prod_{t<i, t\in U_j} \frac{b_{tj,-n}^{(z)}}{a+n_{j,-n}^{(t)}+b_{tj,-n}^{(z)}} \cdot \frac{m_{j,-n}^{(d)}+\alpha}{m_{.,-n}^{(d)}+K\alpha} & \text{if } u_{1_j} < i < u_{L_j} \\
\prod_{t<i, t\in U_j} \frac{b_{tj,-n}^{(z)}}{a+n_{j,-n}^{(t)}+b_{tj,-n}^{(z)}} \cdot \frac{m_{j,-n}^{(d)}+\alpha}{m_{.,-n}^{(d)}+K\alpha} & \text{if } i = u_{L_j} \\
0 & \text{if } i \notin U_j
\end{cases}
$$

1.3.2 Gibbs Sampling of $P(\Delta_{ij} = 1|\boldsymbol{\Delta}_{-i}, \mathbf{w}, \mathbf{z})$

$$
\begin{aligned}
P(\Delta_{ij} = 1|\boldsymbol{\Delta}_{-i}, \mathbf{w}, \mathbf{z}) &= \frac{P(\Delta_{ij} = 1, \boldsymbol{\Delta}_{-i}, \mathbf{w}, \mathbf{z})}{P(\boldsymbol{\Delta}_{-i}, \mathbf{w}, \mathbf{z})} \\
&= \frac{P(\mathbf{w}|\mathbf{z}, \Delta_{ij} = 1, \boldsymbol{\Delta}_{-i})P(\mathbf{z})P(\Delta_{ij} = 1, \boldsymbol{\Delta}_{-i})}{P(\mathbf{w}|\mathbf{z}, \boldsymbol{\Delta}_{-i})P(\mathbf{z})P(\boldsymbol{\Delta}_{-i})} \\
&= \frac{P(\mathbf{w}|\mathbf{z}, \Delta_{ij} = 1, \boldsymbol{\Delta}_{-i})P(\Delta_{ij} = 1, \boldsymbol{\Delta}_{-i})}{P(\mathbf{w}|\mathbf{z}, \boldsymbol{\Delta}_{-i})P(\boldsymbol{\Delta}_{-i})} \\
&\propto P(\mathbf{w}|\mathbf{z}, \Delta_{ij} = 1, \boldsymbol{\Delta}_{-i})P(\Delta_{ij} = 1, \boldsymbol{\Delta}_{-i}) \\
&= \begin{cases}
0 & \text{if } n_j^{(i)} > 0 \\
\pi_{ij} \prod_{k=1}^{K} \left\{ \prod_{l\in U_j,-i} \frac{B(a_{lk}^{(z)}, b_{lk}^{(z)})}{B(a,b)} \right\} \left\{ \sum_{\Delta} \prod_{l=1,-i}^{V-1} \pi_{lk}^{\Delta_{lk}} (1-\pi_{lk})^{(1-\Delta_{lk})} \right\} & \text{if } n_j^{(i)} = 0
\end{cases}
\end{aligned}
$$

$$P(\Delta_{ij}=0|\boldsymbol{\Delta_{-i}},\mathbf{w},\mathbf{z}) \propto P(\mathbf{w}|\mathbf{z},\Delta_{ij}=0,\boldsymbol{\Delta}_{-i})P(\Delta_{ij}=0,\boldsymbol{\Delta}_{-i})$$
$$=(1-\pi_{ij})\frac{B(a_{ij}^{(z)},b_{ij}^{(z)})}{B(a,b)}\prod_{k=1}^{K}\left\{\prod_{l\in U_j,-i}\frac{B(a_{lk}^{(z)},b_{lk}^{(z)})}{B(a,b)}\right\}\left\{\sum_{\Delta}\prod_{l=1,-i}^{V}\pi_{lk}^{\Delta_{lk}}(1-\pi_{lk})^{(1-\Delta_{lk})}\right\}$$

Putting these two results together gives:

$$P(\Delta_{ij}=1|\boldsymbol{\Delta}_{-i},\mathbf{w},\mathbf{z}) = \begin{cases} 0 & \text{if } n_j^{(i)}>0 \\ \frac{\pi_{ij}}{\pi_{ij}+(1-\pi_{ij})\frac{B(a_{ij}^{(z)},b_{ij}^{(z)})}{B(a,b)}} & \text{if } n_j^{(i)}=0 \end{cases}$$

1.4 Estimating β and θ

Both $\boldsymbol{\beta}$ and $\boldsymbol{\theta}$ can be estimated using the predictive distribution over taxon and subcommunity assignments of new sequencing reads.

$$\hat{\beta}_{ij} = Pr(w_{d,new}^{(i)}|z_{d,new}^{(i)}=j,\mathbf{w},\mathbf{z},\boldsymbol{\Delta})$$
$$= \int \underbrace{Pr(w_{d,new}^{(i)}|\boldsymbol{\beta},z_{d,new}^{(i)}=j)}_{\beta_{ij}}\underbrace{Pr(\boldsymbol{\beta}|\mathbf{w},\mathbf{z},\boldsymbol{\Delta})}_{posterior}d\boldsymbol{\beta}$$
$$= \int \beta_{ij}\prod_{l=1_j}^{L_j-1}\frac{1}{B(a_{ij}^{(z)},b_{ij}^{(z)})}\beta_{u_lj}^{a_{u_lj}^{(z)}-1}(1-\beta_{u_{1_j}j}-\cdots-\beta_{u_lj})^{c_{u_l}}d\boldsymbol{\beta}^{(j)}$$

$$\hat{\beta}_{ij} = \begin{cases} \frac{a+n_j^{(i)}}{a+n_j^{(i)}+b_{ij}^{(z)}} & \text{if } i=u_{1_j} \\ \frac{a+n_j^{(i)}}{a+n_j^{(i)}+b_{ij}^{(z)}}\prod_{t<i,t\in U_j}\frac{b_{tj}^{(z)}}{a+n_j^{(t)}+b_{tj}^{(z)}} & \text{if } u_{1_j}<i<u_{L_j} \\ \prod_{t<i,t\in U_j}\frac{b_{tj}^{(z)}}{a+n_j^{(t)}+b_{tj}^{(z)}} & \text{if } i=u_{L_j} \\ 0 & \text{if } i\notin U_j \end{cases}$$

It should be noted that the estimate of β_{ij} does not include any function of π. Any function of π is only introduced via the density of $\boldsymbol{\Delta}$, $Pr(\boldsymbol{\Delta}|\pi)$, which is not needed here since the predictive probability is conditional of $\boldsymbol{\Delta}$.

$$
\begin{aligned}
\hat{\theta}_{dj} &= P(z_{d,new} = j|\mathbf{z}) \\
&= \int \underbrace{Pr(z_{d,new} = j|\boldsymbol{\theta}^{(d)})}_{\theta_{dj}} \underbrace{Pr(\boldsymbol{\theta}^{(d)}|\mathbf{z})}_{posterior} d\boldsymbol{\theta}^{(d)} \\
&= \int \theta_{dj} \frac{\Gamma(m_{\cdot}^{(d)} + K\alpha)}{\prod_{j=1}^{K} \Gamma(m_j^{(d)} + \alpha)} \prod_{k=1}^{K} \theta_{dk}^{m_k^{(d)} + \alpha - 1} d\boldsymbol{\theta}^{(d)} \\
&= \frac{\Gamma(m_{\cdot}^{(d)} + K\alpha)}{\Gamma(m_j^{(d)} + \alpha)} \frac{\Gamma(m_j^{(d)} + \alpha + 1)}{\Gamma(m_{\cdot}^{(d)} + K\alpha + 1)} \\
\hat{\theta}_{dj} &= \frac{m_j^{(d)} + \alpha}{m_{\cdot}^{(d)} + K\alpha}
\end{aligned}
$$

2 SUPPLEMENTARY TABLES AND FIGURES

2.1 Figures

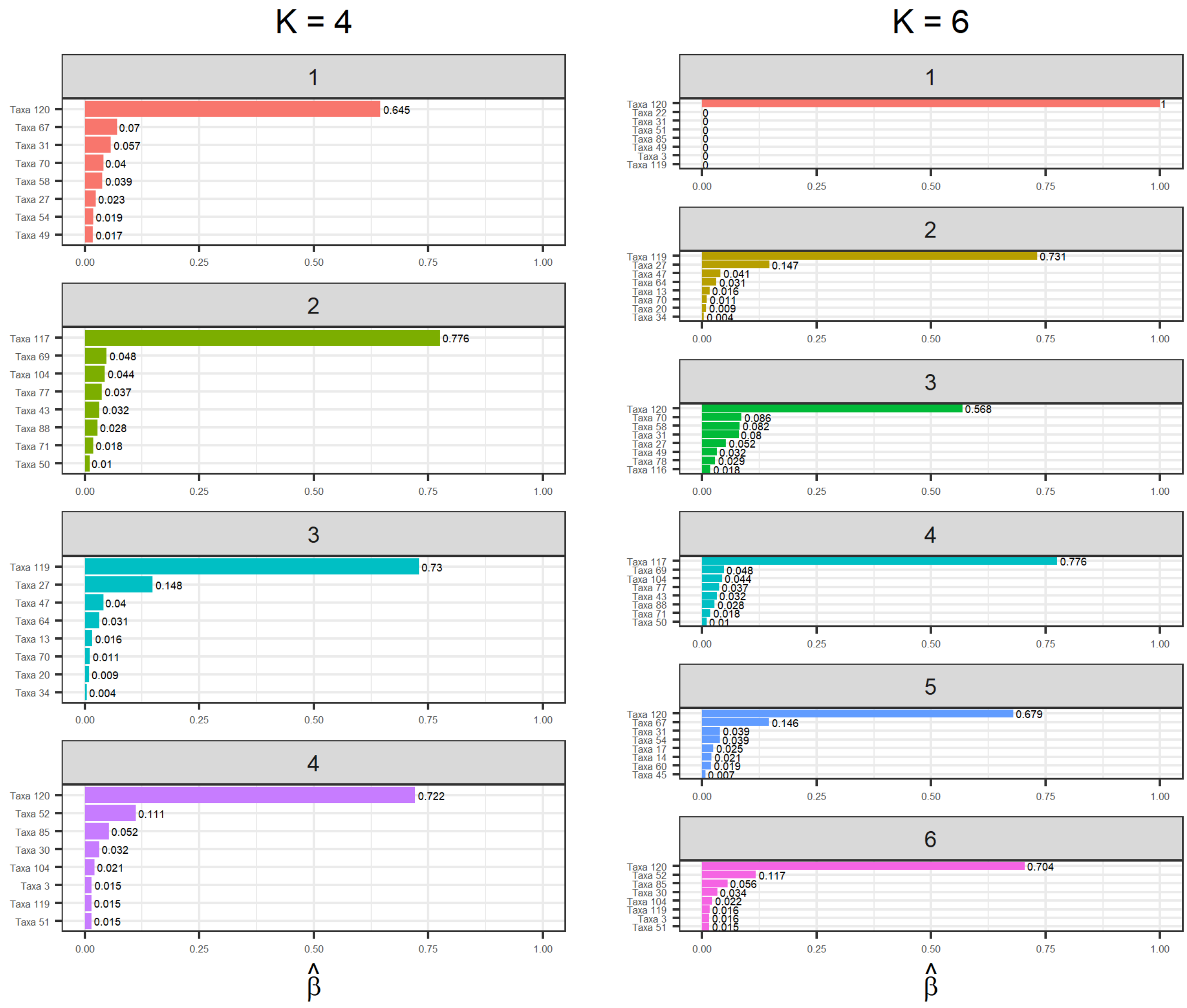

Figure S1. Bar graphs of the top eight taxa, for each subcommunity, with their corresponding β_{ij} values under model misspecification. Data was simulated under a true zero-inflated latent Dirichlet allocation model with five communities and observed V = 87. An underspecified model with four (left) and an overspecified model with six (right) subcommunities were fit to the data.

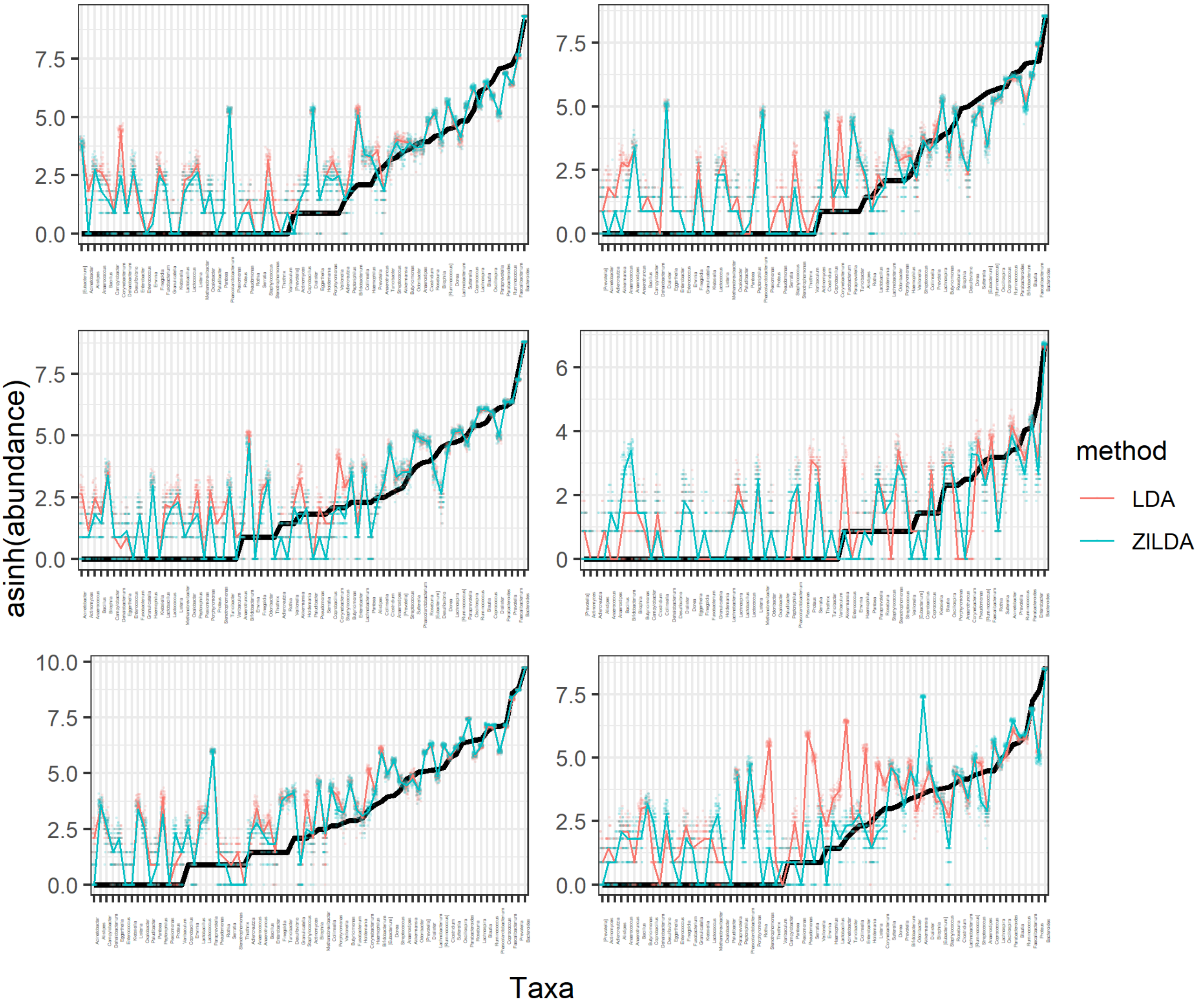

Figure S2. Observed and posterior predictive simulated $asinh$-transformed taxon counts plotted in order of increasing observed abundance from the American Gut Project. Each panel is a different biological sample. The solid black line represents the observed counts. The pink and blue points are the counts from 50 posterior predictive simulated data sets and the pink and blue solid line represents the median counts across all 50 data sets, from the LDA and zinLDA models, respectively.

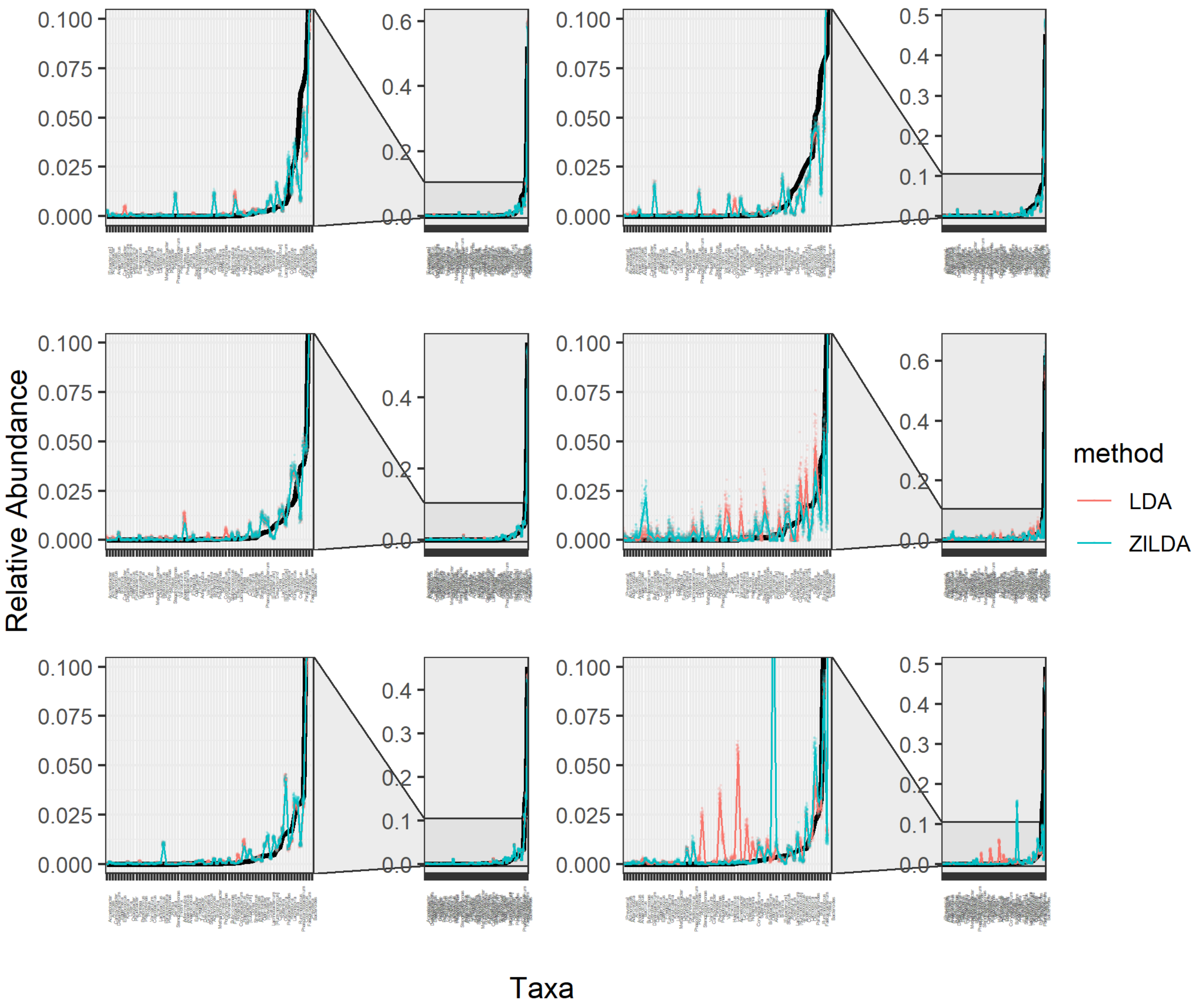

Figure S3. Observed and posterior predictive simulated relative abundances of taxa from the same six samples selected in Figure S2, plotted in order of increasing observed abundance from the American Gut Project. The solid black line represents the observed relative abundance. The pink and blue points are the relative relative abundances from 50 posterior predictive simulated data sets and the pink and blue solid line represents the median abundances across all 50 data sets, from the LDA and zinLDA models, respectively. The first and third column are zoomed-in versions of columns two and four, respectively, showing relative abundances between 0 and 0.1 only.

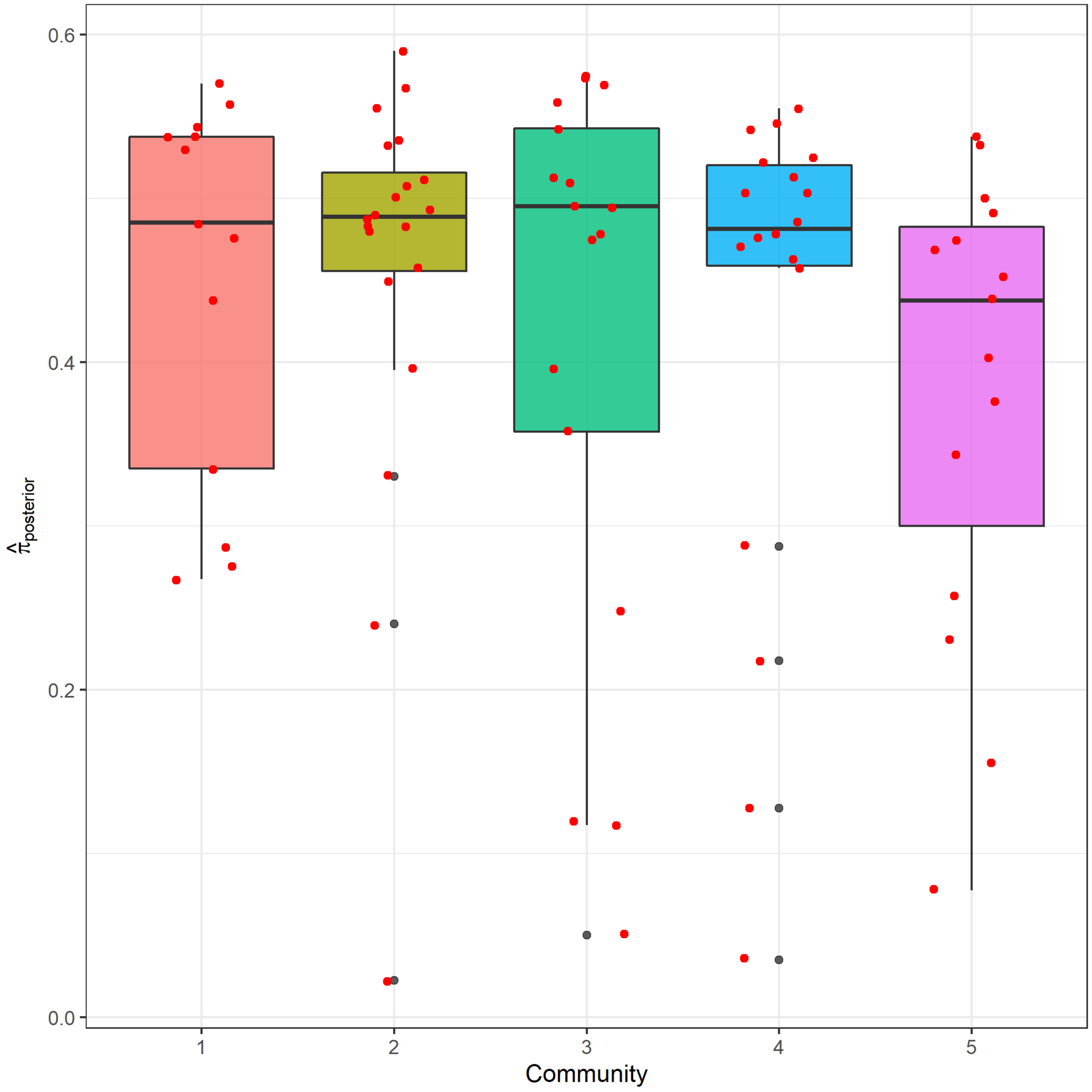

Figure S4. Boxplot of the posterior estimates of π_{ij} for taxa with zero counts split by subcommunity from the zinLDA model applied to a subset of 1000 subjects from the American Gut Project.
